# Berberine Ameliorates Dextran Sulfate Sodium-Induced Ulcerative Colitis and Inhibits the Secretion of Gut Lysozyme via Promoting Autophagy

**DOI:** 10.3390/metabo12080676

**Published:** 2022-07-23

**Authors:** Xiaofan Xu, Wei Li, Zhendong Yu, Le Zhang, Ting Duo, Ya Zhao, Wenxia Qin, Wenbo Yang, Libao Ma

**Affiliations:** Department of Animal Nutrition and Feed Science, College of Animal Science and Technology, Huazhong Agricultural University, Wuhan 430070, China; xiaofan.xu@connect.polyu.hk (X.X.); hzau1898@gmail.com (W.L.); zdyhzau@gmail.com (Z.Y.); liwei230834@gmail.com (L.Z.); duoting0603@webmail.hzau.edu.cn (T.D.); zhaoya.hzau.edu.cn@webmail.hzau.edu.cn (Y.Z.); or 18838945260@163.com (W.Q.); yangwenbo@webmail.hzau.edu.cn (W.Y.)

**Keywords:** berberine, ulcerative colitis, autophagy, lysozyme, inflammation

## Abstract

Ulcerative colitis (UC) is one of the primary types of inflammatory bowel disease, the occurrence of which has been increasing worldwide. Research in recent years has found that the level of lysozyme in the feces and blood of UC patients is abnormally elevated, and the bacterial product after the action of lysozyme can be used as an agonist to recognize different cell pattern receptors, thus regulating the process of intestinal inflammation. Berberine (BBR), as a clinical anti-diarrhea and anti-inflammatory drug, has been used in China for hundreds of years. In this study, results showed that BBR can significantly inhibit the expression and secretion of lysozyme in mice. Therefore, we try to investigate the mechanism behind it and elucidate the new anti-inflammatory mechanism of BBR. In vitro, lipopolysaccharide (LPS) was used to establish an inflammatory cell model, and transcriptomic was used to analyze the differentially expressed genes (DEGs) between the LPS group and the LPS + BBR treatment group. In vivo, dextran sulfate sodium salt (DSS) was used to establish a UC mice model, and histologic section and immunofluorescence trails were used to estimate the effect of BBR on UC mice and the expression of lysozyme in Paneth cells. Research results showed that BBR can inhibit the expression and secretion of lysozyme by promoting autophagy via the AMPK/MTOR/ULK1 pathway, and BBR promotes the maturation and expression of lysosomes. Accordingly, we conclude that inhibiting the expression and secretion of intestinal lysozyme is a new anti-inflammatory mechanism of BBR.

## 1. Introduction

Inflammatory bowel diseases (IBD) can be divided into two types, ulcerative colitis (UC) and Crohn’s disease (CD), and are multifactorial, chronic diseases [1], which affect millions of people all around the world every year. The clinical symptoms of IBD include diarrhea, abdominal pain, and even bloody stools [2]. UC is a chronic, non-specific inflammatory colorectal disease whose etiology is not well understood [3]. Dextran sulfate sodium salt (DSS)-induced UC mice models have been widely used in UC research. The symptoms and histologic changes are similar to those of human UC [4]. Therefore, we used the DSS-induced intestinal inflammation mouse model to study the pharmacological mechanism of berberine (BBR). 

BBR is an isoquinoline alkaloid found in many types of medicinal plant, including the families Papaveraceae, Berberidaceae, Fumariaceae, Menispermaceae, Ranunculaceae, Rutaceae, and Annonaceae. These herb plants have been clinically used for thousands of years in China to treat gastrointestinal diseases [5]. BBR has been used to treat diabetes and hyperlipidemia in recent years because of its excellent anti-inflammatory and anti-bacterial effects [6]. Research has shown that BBR’s ability to improve hyperglycemia might be attributed to increased microbiome-mediated deoxycholic acid (DCA) production, which up-regulates colonic expression of Takeda G protein-coupled receptor 5 (TGR5) and the secretion of glucagon-like peptide (GLP) and improves glucose, lipid, and energy metabolism in db/db mice [7]. BBR as an anti-inflammatory drug candidate has demonstrated excellent efficacies in various in vitro or in vivo studies. Previous studies have suggested that BBR exhibits anti-hepatoma activity by inducing apoptotic, necrotic, and autophagic cell deaths in various cell types [8,9,10], can treat neurogenic inflammatory disease [11], and enhances intestinal mucosal immunity [7] and intestinal epithelial barriers [12]. In addition, some studies have indicated that BBR is a regulator of autophagy, which can influence the progress of some diseases. 

Autophagy is a process of intracellular self-degradation responsible for the removal, degradation, and recycling of cytoplasmic proteins and organelles [13]. Through continuous research, scholars have discovered that there is a very close relationship between intestinal epithelial cell autophagy and intestinal inflammation [14,15]. Although inflammatory response can protect our body from damage and repair tissue after infection, it may cause irreversible damage if our body is constantly in the process of inflammatory response [16]. Previous studies have found that autophagy can significantly inhibit the inflammatory response of cells [17,18]. The underlying mechanism may be directly inhibiting the formation of inflammatory complexes or indirectly alleviating the stimulation caused by inflammation, thereby protecting our cells from excessive and continuous inflammatory damage [19]. 

Paneth cells (PCs) are important cells in the small intestine that provide the host with a defense against microbial invasion [20,21]. When bacteria or bacterial antigens invade the body, PCs secrete antibacterial molecules such as defensins and lysozyme into the villi of the lumen of the small intestine to help maintain the gastrointestinal barrier [22]. Several UC-associated genes such as autophagy related protein 16 like protein (ATG16L 1), nucleotide-binding oligomerization domain (NOD 1), and nucleotide-binding oligomerization domain (NOD 2) are highly expressed in the PCs, and these genes can influence the function of PCs as well, which indicates that PCs may play a vital role during the process of UC [23]. At present, many important cellular activities in PCs, such as autophagy and the regulation of lysozyme, have not been fully understood yet. The abnormal expression or release of lysozyme is considered one of the pathological features of PCs [24]. Lysozyme is an important antimicrobial substance in the innate immune system of animals [25]. In addition to its direct bacteriostatic action, lysozyme also has other biological functions, such as regulating tumor necrosis factor alpha (TNF-α) signaling pathways [26]. The product of lysozyme treatment of bacteria, such as muramyl dipeptide (MDP), lipopolysaccharide (LPS), and γ-d-glutamyl-meso-diaminopimelic acid (iE-DAP), can be used as agonists to recognize different pattern receptors, notably NOD-like receptors (NLR) and toll-like receptors (TLR) [27], which can regulate intestinal inflammation. 

Our findings showed that BBR can inhibit the expression and secretion of lysozyme by promoting autophagy via adenosine 5‘-monophosphate (AMP)-activated protein kinase (AMPK)/mammalian target of rapamycin (MTOR)/unlike autophagy activating kinase 1(ULK1) pathway, and BBR promotes the maturation and expression of lysosomes. Therefore, BBR can be a candidate drug to treat UC patients in the future.

## 2. Materials and Methods

### 2.1. Trial 1: Therapeutic Effect of Berberine on DSS-Induced Enteritis in Mice

#### 2.1.1. Experimental Animals and Grouping

Kunming mouse is a commonly used animal in laboratories. It has a stable genetic background and strong adaptability. Female Kunming mice have a mild personality, and, to exclude the factor of gender differences, Specific Pathogen Free (SPF) female Kunming mice were used in all animal trials in this experiment. 

Kunming mice (8 weeks old, female) with similar body weight (about 25 g) were selected for the experiment. According to previous research, we have set three different concentrations of BBR [28]. A total of 120 Kunming female mice were randomly divided into six groups: control group, DSS group, DSS + normal saline (NS) group, DSS + 25 mg/kg BBR, DSS + 50 mg/kg BBR, and DSS + 100 mg/kg BBR, with five replicates per group and four mice per replicate. After 3 days of environmental adaptation, the first group (control group) was fed normally, and the second group (DSS group) drank water containing 3% DSS, respectively, for 7 d, then the mice were killed; the third group (DSS + NS group) was fed water containing 3% DSS for 7 d and then normal saline for 5 d, and the mice were killed; groups 4–6 (DSS + BBR groups) were all fed 3% DSS water for 7 d, then administered with 25 mg/kg, 50 mg/kg, and 100 mg/kg BBR, respectively, for 5 d, and the mice were killed.

#### 2.1.2. Intestinal Sample Collection and Treatment

After the mice were killed, the colon and ileum of the mice were collected, washed with phosphate buffered saline (PBS) to remove the contents of the intestine, and divided into two parts. One part was fixed with 4% paraformaldehyde, and the intestinal morphology and immunofluorescence analyses were performed. The other part was stored in −80 °C refrigerator, prepared for protein and RNA expression analysis.

#### 2.1.3. Mice Body Weight Data Collection

During the experiment, the weight of each mouse was measured every morning for 12 days. The weight of each cage of mice was calculated based on the average body weight of four mice in each cage.

#### 2.1.4. Statistics of Mice Disease Activity Index

Disease activity index (DAI) is a comprehensive score that combines the percentage of diseased animals’ weight loss, stool viscosity, and stool bleeding. The total score of the three results is divided by 3 to obtain the DAI score. The specific scoring standards are shown in Supplementary Table S1.

#### 2.1.5. Mice Colon Tissue Injury Score

The colon of the mice in each treatment group was scored for tissue damage, and the average score of four mice in each cage was calculated. The specific scoring standards are shown in Supplementary Table S2.

#### 2.1.6. Intestinal Tissue Immunofluorescence Test

The ileum was flushed and embedded in paraffin. The fragments were cut into approximately 5 mm-thick sections and stained with 5 mg/mL wheat germ agglutinin (WGA) for 10 min at room temperature. Subsequently, the slides were permeated with PBS buffer containing 0.2% Triton X-100 for 15 min at room temperature, and then blocked with PBS buffer containing 5% BSA at room temperature for 1 h. The slides were incubated with the primary Lyz1 antibody overnight at 4 °C, and then with the secondary antibody for 1 h at room temperature. The slides were incubated with PBS buffer containing 1 mg/mL DAPI solution for nuclear staining. Finally, the slides were checked with a fluorescence microscope.

#### 2.1.7. Intestinal Tissue Immunofluorescence Data Statistics 

Lysozyme immunofluorescence tests were performed on the ileum of mice in each treatment group, and the number of lysozyme-positive crypts in the ileum of the mice was counted. The average number of four mice per cage was used for calculation. 

#### 2.1.8. Intestinal Lysozyme ELISA Assay

Intestinal lysozyme levels were determined using the ELISA method. ELISA kits from LifeSpan BioSciences company (LS-F24252) were used to measure intestinal lysozyme levels according to the instructions. The absorbance at 450 nm was determined using a microplate reader (Perkin Elmer/Wallac Victor 2 1420-012 system).

### 2.2. Transcriptome Analysis of the IEC-18 Inflammatory Model Induced by LPS

#### 2.2.1. Cell Grouping and Sample Collection

Normally adherent IEC-18 cells were divided into four cell test groups (control group, LPS group, LPS + BBR group, and LPS + DMSO group), with three replicates for each treatment. The control group cells were cultured in normal medium for 12 h, and then total RNA was collected from the cells with TRIZOL reagent. Cells in the LPS group were cultured in a medium containing LPS (10 μg/mL) for 12 h, and total cellular RNA was collected. Cells in the LPS + BBR group were cultured in LPS (10 μg/mL) culture medium for 12 h, then in BBR (100 μM) culture medium for 24 h, and total cell RNA was collected. Cells in the LPS + DMSO group were cultured in a medium containing LPS (10 μg/mL) for 12 h, then in a medium containing DMSO (0.2%) for 24 h, and total cell RNA was collected. BBR is soluble in DMSO (0.2%). In all experiments, IEC-18 cells were cultured in DMEM medium containing 10% fetal bovine serum (FBS), 1% penicillin/streptomycin, and 0.1 µg/mL insulin at a culture temperature of 37 °C and a carbon dioxide concentration of 5%. TRIzol reagent was used to extract total RNA from cells and DNase I was used to remove genomic DNA. Finally, an Agilent 2100 bioanalyzer was used to determine the quality of RNA, and a Nanidrop 2000 instrument was used to quantify.

#### 2.2.2. Illumina Hseq4000 Sequencing

The preparation of the RNA-seq transcriptome library follows the requirements of TruSeq™ Illumina’s RNA sample preparation kit, using 5 μg total RNA. An amount of 200–300 bp cDNA target fragment library was selected with 2% low-range ultra-agarose, and then Phusion DNA polymerase was used to carry out 15 PCR cycles of PCR amplification. After quantification using a TBS380 mini fluorometer, the paired-end RNA-seq sequencing library was sequenced using an Illumina HiSeq 4000 system (2 × 150 bp read length).

#### 2.2.3. Transcriptome Data Analysis

The expression level of each transcript was calculated based on the number of fragments per kilobase of exons/1 million mapped reads (FRKM). RNA sequence expectation maximization (RSEM) (http://deweylab.biostat.wisc.edu/rsem/ (accessed on 7 February 2020)) was used to quantify gene abundance. Differentially expressed genes (DEG) were determined by pairwise comparison using EdgeR (empirical analysis of digital gene expression in R). In the four control groups (i.e., control group and LPS; LPS and LPS + BBR; LPS and LPS+ In DMSO; and LPS + BBR and LPS + DMSO), genes with fold change ≥ 2 and *p* < 0.05 abundance were considered to be differently regulated. To further study the biological processes related to DEGs, GO analysis was performed by running a query on each DEG against the GO database. The GO database provides information about molecular functions, cellular components, and biological processes. Subsequently, KEGG functional enrichment analysis was performed to identify DEGs that were significantly enriched in the anti-inflammatory pathway compared with the background of the entire transcriptome (*p* ≤ 0.05). Metaboanalyst4.0 online software was used for principal component analysis (PCA) and hierarchical cluster analysis (HCA) to evaluate the similarity and difference of transcriptome profiles.

### 2.3. Trial 3: The Effect of Berberine on the Level of IEC-18 Autophagy and Lysosome

#### 2.3.1. Autophagy Fluorescent Staining with MDC

Normally adherent IEC-18 cells were divided into six groups (control group, LPS group, LPS + DMSO group, LPS + 25 μM BBR group, LPS + 50 μM BBR group, and LPS + 100 μM BBR group), three replicates for each treatment. The concentration was determined based on previous studies [29]. Cells in the control group and in the LPS group were cultured in normal medium and a medium containing LPS (10 μg/mL), respectively, for 12 h, and then stained. The three groups of BBR-treated cells were cultured in LPS (10 μg/mL) medium for 12 h, and then in BBR (25 μM, 50 μM, 100 μM) medium for 24 h before staining. The cells in all groups were treated with 4% paraformaldehyde. After the cells were washed with PBS, they were stained with monodansylcadaverine (MDC) for 30 min and observed with a fluorescence microscope.

#### 2.3.2. Western-Blot Analysis

Total protein was extracted from the above six groups of cells, and a specific Western blot test was performed to detect the expression levels of AMPK, MTOR, ULK1, and Light chain 3 beta (LC3B) proteins in different treatment groups. Lysis buffer (50 mM Tris, 150 mM NaCl, 1 mM EDTA, 1% Triton X-100, 1 μg/mL pepsin inhibitor, 1 μg/mL aprotinin, 1 μg/mL leucin, and 1 mM PMSF) was used to prepare whole cell lysates of cells for Western blotting. First, the protein extract was subjected to sodium dodecyl sulfate-polyacrylamide gel electrophoresis (SDS-PAGE) with a 6% or 12% polyacrylamide gel, and then transferred to a polyvinylidene fluoride (PDVF) membrane. Subsequently, the PVDF membrane was incubated in Tris-buffered saline (TBS) buffer containing 0.1% Tween and 5% fat-free milk for 1 h, and then with specific primary antibodies overnight at 4 °C. The PVDF membrane was incubated with the secondary antibody bound to horseradish peroxidase (HRP) for 1.5 h at room temperature. Finally, an enzyme-linked enhanced chemiluminescence (ECL) reagent was used to detect the signal.

#### 2.3.3. Cell Lysosome Fluorescence Staining

We used lysosomes specifically labeled Lamp-1 to label the lysosomes of IEC-18 cells for immunofluorescence detection and counted the number of lysosomes in different treatment groups. The above six groups of cells (three repetitions in each group) were washed three times with PBS for 3 min each, followed by fixing the slides with 4% paraformaldehyde for 15 min and washing the slides with PBS three times. Next, 0.5% Triton X-100 (prepared in PBS) was permeabilized at room temperature for 20 min. Normal goat serum was dropped on the slide and blocked for 30 min at room temperature. The blocking solution was absorbed with absorbent paper, and enough diluted LAMP1 primary antibody was added to each slide, which was then placed in a humidified box and incubated overnight at 4 °C. The next day, the slide was soaked with PBST three times for 3 min each time. The excess liquid on the slide was absorbed by absorbent paper, and diluted fluorescent secondary antibody was added dropwise to the slide, which was then incubated in a humid box at room temperature for 1 h and soaked with PBST three times for 3 min each time. Finally, the slide was mounted with mounting solution containing anti-fluorescence quencher. It was observed and the image was collected under a fluorescence microscope.

#### 2.3.4. Real-Time Fluorescence Quantitative PCR

The key genes in the AMPK/MTOR/ULK1 pathway of the above six groups of cells (three replicates in each group) were detected by real-time fluorescent quantitative PCR. We selected 11 specific genes for PCR detection, with GAPDH as the internal reference gene.

#### 2.3.5. Primer Synthesis

The primer’s sequence for each target gene and reference gene are from PrimerBank, and primers are synthesized by Shenggong Technology (Shanghai, China). The primers for each gene are shown in Supplementary Table S3.

#### 2.3.6. Data Analysis

The results were expressed as mean ± SEM. The experimental data were analyzed by one-way analysis of variance, and then Duncan’s multiple comparison test was performed using GraphPad 8.0 software (GraphPad software, Inc., Beijing, China). A value of *p* < 0.05 is considered a statistically significant difference.

### 2.4. Trial 4: The Effect of Berberine and Autophagy Promoters/Inhibitors on DSS-Induced Enteritis and Lysozyme Secretion in Mice

#### 2.4.1. Experimental Animals and Grouping

Kunming mice (8 weeks old, female) with similar body weight (about 25 g) were selected for the experiment. A total of 140 Kunming female mice were randomly divided into seven groups. The mice in the same group were randomly assigned to five cages, four in each cage. The first group (control group) was fed normally for 12 d. Group 2 (DSS group) drank water containing 3% DSS for 7 d, and all mice were killed. The third group (DSS + NS) was given water containing 3% DSS for 7 d and then normal saline for 5 days, and the mice were sacrificed. The fourth group (DSS + BBR) was given water containing 3% DSS by gavage for 7 d and then 50 mg/kg BBR normal saline for 5 d, and the mice were sacrificed. Group 5 (DSS + RAPA) drank water containing 3% DSS for 7 d, and was intraperitoneally injected with 2.0 mg/kg rapamycin (RAPA) for 5 d, following the method described in a previous article [30], then the mice were sacrificed. Group 6 (DSS + BBR + 3-MA) drank water containing 3% DSS for 7 d, and then was given 50 mg/kg BBR and intraperitoneally injected with 15 mg/kg 3-methyladenine (3-MA) for 5 d, following the method described in a previous article, and the mice were sacrificed. Group 7 (DSS + 3-MA) was given water containing 3% DSS for 7 d, and intraperitoneally injected with 15 mg/kg 3-methyladenine (3-MA) for the next 5 d, then the mice were sacrificed.

#### 2.4.2. Morphological Analysis of Intestinal Tissue

The colon and ileum were taken out of the 4% paraformaldehyde solution, cleaned, and then embedded in paraffin. Subsequently, these segments were sliced into approximately 5 μm-thick sections, which were first stained with hematoxylin and then with eosin. Digital images of the intestinal morphology at 10 times, 40 times, and 100 times magnification were obtained using an optical microscope. Using Image-Pro-Plus software (version 6.0), based on at least 30 representative and well-positioned villi and crypts per mouse, the intestinal villi height and crypt depth were measured blindly. For each intestinal segment, the villus height is defined as the distance from the crypt–villi junction to the top of the villi, and the crypt depth is defined as the distance from the bottom of the crypt to the crypt–villi junction.

#### 2.4.3. ELISA Detection of Intestinal Inflammatory Cytokines

In order to detect the intestinal inflammation level of mice in each treatment group, we used ELISA to detect the levels of two intestinal pro-inflammatory cytokines (TNF-α and IL-17A) and two anti-inflammatory cytokines (IL-4 and IL-13). The tissue was cut into pieces. The minced tissues were homogenized with pre-cooled PBS (containing protease inhibitors) in a certain proportion and centrifuged (4 °C, 5000× *g* for 5–10 min), and then the supernatant was taken for experiment. Each group had three replicates. The experimental procedures were carried out according to the product instructions.

#### 2.4.4. Intestinal Tissue Immunofluorescence Test

The ileum segments of the six groups of mice were washed and embedded in paraffin. The fragments were cut into approximately 5 mm-thick sections and stained with 5 mg/mL wheat germ agglutinin (WGA) for 10 min at room temperature. Subsequently, the slides were permeated with PBS buffer containing 0.2% Triton X-100 at room temperature for 15 min, and then blocked with PBS buffer containing 5% BSA at room temperature for 1 h. The slides were incubated overnight with primary Lyz1 and LC3B antibodies at 4 °C, and then incubated with secondary antibodies for 1 h at room temperature. The slides were incubated with PBS buffer containing 1 mg/mL DAPI solution for nuclear staining. Finally, the slides were checked with a fluorescence microscope.

#### 2.4.5. Statistics of Intestinal Immunofluorescence Data

After performing intestinal immunofluorescence on the above six groups of mice, the number of lysozyme-positive crypts, the number of Paneth cells in the positive crypts, and the LC3B fluorescence expression in the lysozyme-positive area of each fluorescent section were counted, and ImageJ was used, with 20 repeats per group. The results are expressed as mean ± SEM. The experimental data were analyzed by one-way analysis of variance, and then Duncan’s multiple comparison test was performed using GraphPad 8.0 software (GraphPad software, Inc. Beijing, China.). A value of *p* <0.05 was considered a statistically significant difference.

## 3. Results

### 3.1. Berberine Ameliorates DSS-Induced Ulcerative Colitis and Inhibits the Expression and Secretion of Lysozyme

**Figure 1:**
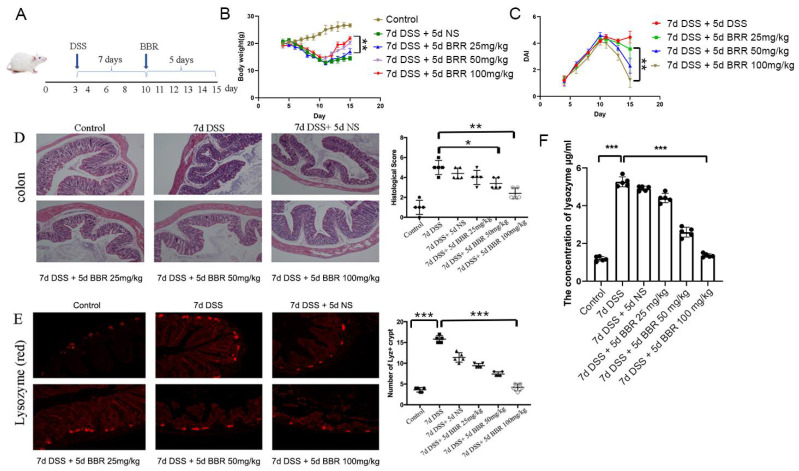
Berberine ameliorates DSS-induced ulcerative colitis and inhibits the secretion of lysozyme. (**A**) Research design: 3% DSS for 7 d and BBR for 5 d; (**B**) daily body weight of mice (*n* = 5); (**C**) disease activity index (DAI) between day 3 and day 15 (*n* = 5); (**D**) hematoxylin-eosin (H-E) histological sections of colon (*n* = 5); (**E**) immunofluorescence images of the lysozyme in the ileum of six groups (*n* = 5); (**F**) detection of the lysozyme concentration in the ileum by ELISA (*n* = 5). * *p* < 0.05, ** *p* < 0.01, *** *p* < 0.001.

The experiment lasted 15 days. The first three days were allowed for the mice to adapt to the environment. All the mice except the control group were then treated with DSS (3%) for 7 d. Subsequently, they were further treated with normal saline (NS), 25 mg/kg BBR, 50 mg/kg BBR, and 100 mg/kg BBR, respectively (Figure 1A). A significant decrease in the body weight of DSS-induced mice was observed from day 4 until day 10, but the body weight began to increase after the treatment of BBR (day 11 to day 15), and the effect depended on the concentration of BBR; the 100 mg/kg group showed significantly increased body weight compared to the 25 mg/kg group (*p* < 0.01) (Figure 1B). Disease activity index (DAI) results indicated that BBR could decrease the DAI of mice significantly (*p* < 0.01), and the effect was concentration-dependent (Figure 1C). Hematoxylin-eosin (H-E) histological section showed that DSS caused serious damage to the colon. After being treated differently for 5 d, the histological scores indicated that damage was not alleviated greatly in the NS group, but it was significantly relieved in the 50 mg/kg BBR group (*p* < 0.05) and the 100 mg/kg (*p* < 0.0.1) group compared to the NS and DSS groups (Figure 1D). The excessive secretion of lysozyme to the intestinal tract usually causes dysbacteriosis and other inflammation problems. Finally, we found that BBR can influence the expression and secretion of lysozyme in mice. Compared to the control group, the expression and secretion of lysozyme in the 7d DSS group increased significantly (*p* < 0.01). The immunofluorescence trial and ELISA showed that BBR reduced lysozyme expression and secretion in Paneth cells significantly (*p* < 0.0001) (Figure 1E,F), and the effect depended on concentration. 

### 3.2. Berberine Improves the Expression of Autophagy-Related Genes Based on Transcriptomic Analysis

**Figure 2:**
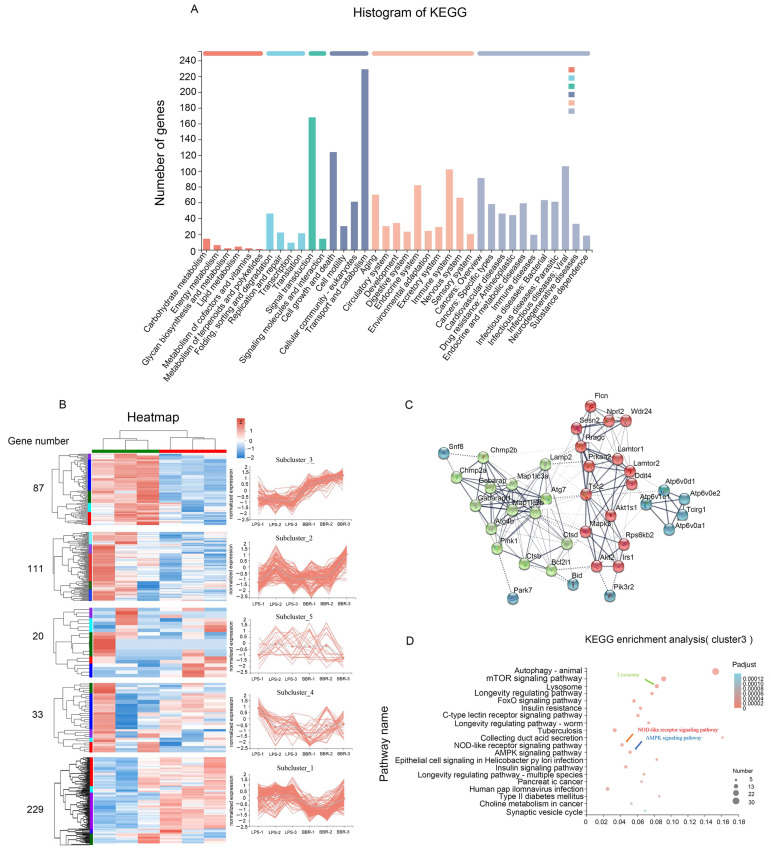
IEC-18 transcriptomic analysis. (**A**) KEGG enrichment analysis of the DEGs between LPS and LPS + BBR groups; (**B**) clustering analysis: divide DEGs grouped into five sub-clusters according to the gene expression trendy; (**C**) protein–protein interaction (PPI) network based on STRING database; (**D**) KEGG enrichment analysis of the genes from sub-cluster 3.

We wanted to determine how BBR reduces the secretion of lysozyme. In vitro, we used LPS to establish an inflammatory cell model and transcriptomics to analyze the differentially expressed genes (DEGs) between LPS and LPS + BBR groups. KEGG enrichment analysis indicated that a total of 480 genes were enriched on the autophagy-associated pathway (Figure 2A). Genes with similar expression patterns usually exhibit related functions. We used the 480 genes to do clustering analysis, and divided them into five sub-clusters based on their expression patterns. In sub-cluster 3, a significant difference between LPS and LPS + BBR groups in gene expression was detected, and such a difference was coherent in all repeats (Figure 2B). The genes involved in sub-cluster 3 were analyzed by protein–protein interaction (PPI) network and KEGG enrichment analysis based on STRING database. Thirty-eight genes showed significant interactions, and we marked three sub-networks with strong interaction with three different colors (Figure 2C). KEGG enrichment analysis showed that DEGs were enriched in the pathways of autophagy, lysosome, NOD-like receptor, and AMPK (Figure 2D).

### 3.3. Berberine Promotes Autophagy through the AMPK/MTOR/ULK1 Pathway

**Figure 3:**
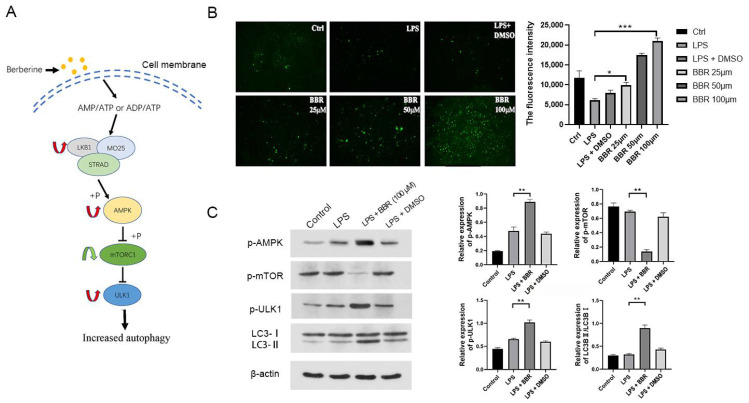
Berberine promotes autophagy through the AMPK/MTOR/ULK1 pathway. (**A**) DEGs between LPS and LPS + BBR groups enriched in autophagy (red arrows indicating the increase of gene expression and green arrows decrease); (**B**) autophagy detected by MDC staining; (**C**) Western-blot images of autophagy-related proteins in four groups (*n* = 3). * *p* < 0.05, ** *p* < 0.01, *** *p* < 0.001.

The results from transcriptomic analysis indicated that BBR regulated autophagy-associated genes. To evaluate the effect of BBR on autophagy, we examined the formation of autophagosomes in IEC-18 cells using MDC staining and examined the expression of the autophagy-associated proteins LC3B by Western blot. We found that the number of MDC foci decreased after treatment with LPS, but the number of MDC foci significantly increased (*p* < 0.001) after treatment with BBR, and the effect of BBR depended on concentration (Figure 3B). The expression levels of LC3, markers of autophagy, is shown in Figure 3C. After treatment with BBR, the expression of LC3B showed obvious elevation compared to that in the LPS groups. These results demonstrated that autophagy activity in IEC-18 cells was increased by treatment with BBR. We checked gene expression with RNA sequencing, and found that BBR promoted autophagy through the AMPK/MTOR/ULK1 pathway (Figure 3A). We also checked the protein expression of these genes and found that BBR significantly increased the expression of p-AMPK (*p* < 0.05), P-ULK1 (*p* < 0.05), and LC3B (*p* < 0.05) and inhibited the expression of p-MTOR (*p* < 0.05) (Figure 3C).

### 3.4. Berberine Alleviates DSS-Induced Ulcerative Colitis and Inhibits the Expression and Secretion of Lysozyme via Promoting Autophagy

**Figure 4:**
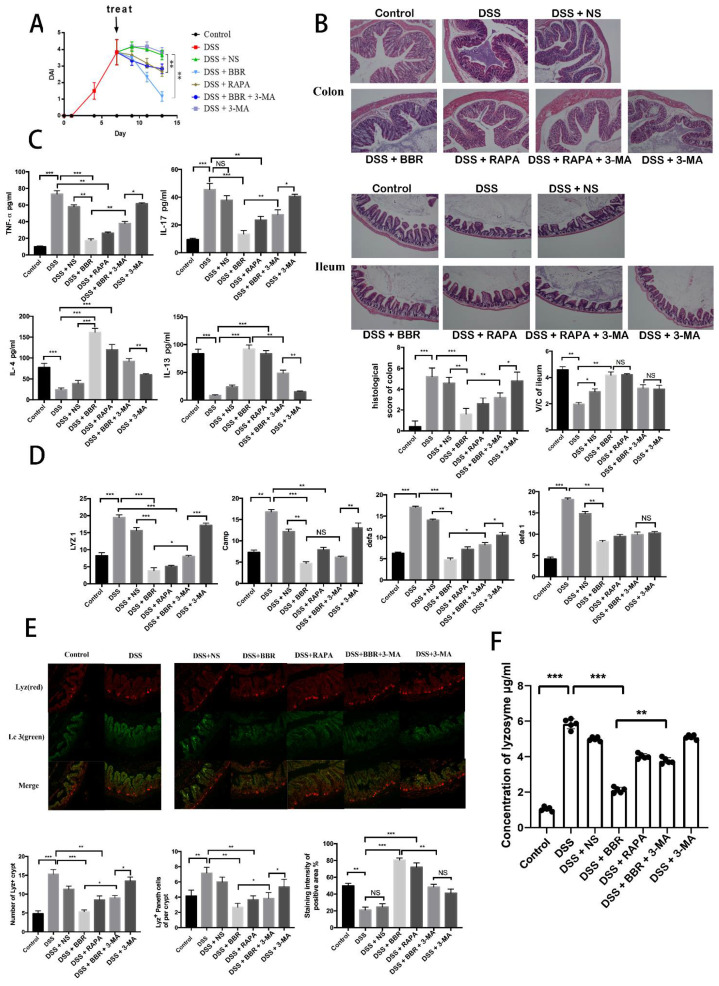
Berberine alleviates DSS-induced ulcerative colitis and inhibits the secretion of lysozyme via promoting autophagy. In these experiments, 100 mg/kg BBR was used to treat the mice. (**A**) Disease activity index (DAI) between day 3 and day 15 (*n* = 5); (**B**) hematoxylin-eosin (H-E) histological sections of colon and ileum (*n* = 5), histological score for colon, the villus height, and crypt depth ratio (V/C) for ileum; (**C**) expression levels of inflammatory factors (*n* = 5); (**D**) gene expression of Lyz1, Camp, Defa1, and Defa5 (*n* = 5); (**E**) immunofluorescence images of lysozyme in the ileum of six groups (red for lysozyme and green for LC3B) (*n* = 5); (**F**) detection of the lysozyme concentration in the ileum by ELISA (*n* = 5). * *p* < 0.05, ** *p* < 0.01, *** *p* < 0.001, NS, Not Significant.

Previously, we found that BBR can alleviate the damage of DSS-induced ulcerative colitis and reduce the secretion of lysozyme (Figure 1D,E). Nevertheless, how does BBR take effect? Does autophagy play an important role in this process? Rapamycin (RAPA) is an inhibitor of mTOR and can promote autophagy; 3-Methyladenine (3-MA) is an inhibitor of PI3K and can inhibit autophagy. We used 3% DSS to induce ulcerative colitis in our mice model, and then the mice were treated with BBR, RAPA, and BBR + 3-MA, respectively. Hematoxylin-eosin (H-E) histological section showed that DSS caused serious damage to the colon and ileum, and after being treated differently for 5 days, the histological score, villus length/crypt depth (V/C), and DAI (Figure 4A,B) indicated that the damage in the NS group was not greatly alleviated, but that in BBR and RAPA groups was significantly relieved (*p* < 0.01). However, combination therapy with 3-MA attenuated the effect of BBR (*p* < 0.01) compared to the group treated only with BBR (Figure 4B). Moreover, we tested two pro-inflammatory cytokines (TNF-α and IL-17A) and two anti-inflammatory cytokines (IL-4 and IL-13) from serum with the ELISA kit. The results showed BBR and RAPA decreased the secretion of TNF-α (*p* < 0.01) and IL-17A (*p* < 0.01) compared to the DSS group and enhanced the production of IL-4 and IL-13. The anti-inflammatory effect of BBR was attenuated by 3-MA compared to the group treated with BBR alone (*p* < 0.01) (Figure 4C).

Lysozyme is mainly secreted by Paneth cells in the intestinal tract. In addition to Lyz1, Paneth cells secreted other AMPs (Camp, Defa5, Defa1) as well. We tested the expression of relevant genes with qPCR. The results indicated that autophagy could regulate the function of Paneth cells (Figure 4D). We collected the ileum from different groups and used Lyz1 antibody and LC3B antibody to indicate the situation of lysozyme and autophagy in immunofluorescence. The Lyz1 (red light) was distributed in crypts. DSS obviously increased the numbers of Lyz+ crypts and Lyz+ Paneth cells per crypt. In contrast, the numbers of Lyz+ crypt and Lyz+ Paneth cells per crypt in BBR and RAPA groups decreased significantly compared with the DSS group, and 3-MA attenuated the effect of BBR. In this trial, we focused on the area that expressed red light strongly, because this area is the location of Paneth cells; we used ImageJ to analyze the expression of green light (LC3B) in this area. The result showed that BBR increased the LC3B+ (green) staining intensity of Lyz+ (red) area, which means that BBR can promote the autophagy of Paneth cells compared with the DSS group (Figure 4E). In addition, we used the ELISA kit to detect the secretion of lysozyme in the ileum. The results showed that BBR can significantly inhibit the secretion of lysozyme compared to the DSS group (*p* < 0.01).

### 3.5. Berberine Promotes the Maturation and Expression of Lysosomes

**Figure 5:**
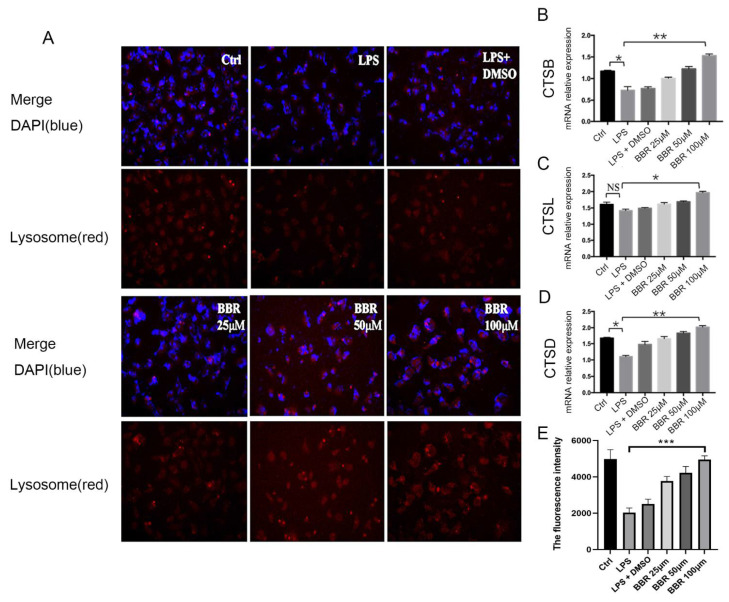
Berberine promotes the maturation and expression of lysosomes. (**A**) Immunofluorescence images of lysosome in IEC-18; (**B**) the fluorescence intensity analysis of the lysosome IF staining; (**C**) CTSB mRNA relative expression; (**D**) CTSL mRNA relative expression; (**E**) CTSD mRNA relative expression. *n* = 5. * *p* < 0.05, ** *p* < 0.01, *** *p* < 0.001, NS, Not Significiant.

Previous research has found that lysozyme is degraded by lysosome in Paneth cells. We found that autophagy can regulate the secretion of lysozyme, and further wanted to figure out whether it is associated with the function of lysosome. We used Lamp-1, a special marker of lysosome, to mark the lysosome in IEC-18 cells. The immunofluorescence experiment showed that BBR enhanced the number of lysosomes in the cells, and the inhibitory effect was significantly enhanced (*p* < 0.05) with the increase of BBR concentration (Figure 5A,B). In addition to the number of lysosomes, we questioned whether BBR could influence the maturation of lysosome. CTSB, CTSD, and CTSL are associated with the maturation of lysosome. We found the expression of these genes were obviously increased after the treatment of BBR (*p* < 0.01) (Figure 5C–E).

### 3.6. Berberine Regulates the ATG16L1/NOD1/RIPK2 Signaling Pathway

**Figure 6:**
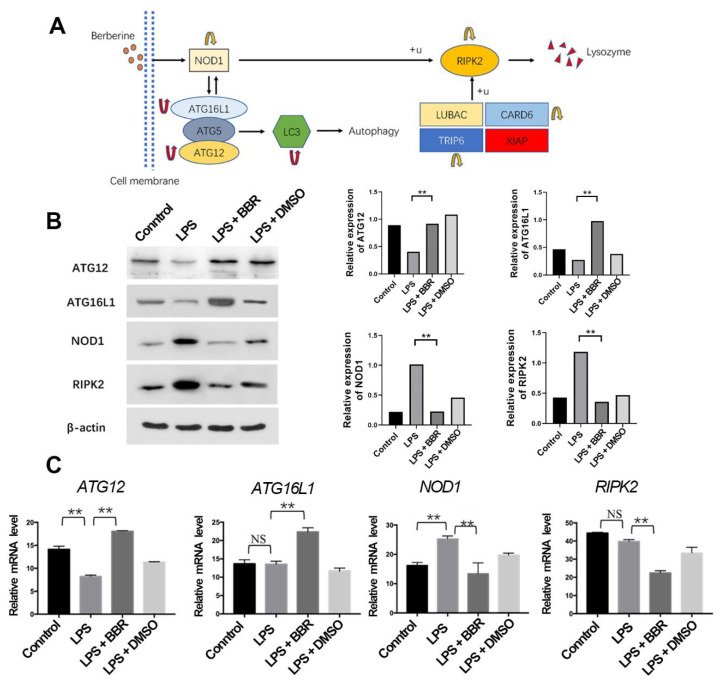
Berberine regulates the ATG16L1/NOD1/RIPK2 signaling pathway. (**A**) DEGs between LPS and LPS + BBR groups enriched in autophagy (red arrows indicate the increase in gene expression and yellow arrows a decrease); (**B**) Western-blot images of ATG12, ATG16L1, NOD1, and RIPK2 in four groups; (**C**) mRNA relative expression of ATG12, ATG16L1, NOD1, and RIPK2 (*n* = 5). ** *p* < 0.01, NS, Not Significiant.

The NLR signaling pathway was reported to be able to influence the downstream inflammatory pathway, and our transcriptomic analysis revealed that many DEGs were enriched on the NOD-like receptor signaling pathway. Atg16L1 and ATG12 are autophagy proteins, and their complex can influence NOD1, and RIPK2 is an important downstream gene of NOD1. We tested the expression of AGT16L1, ATG12, NOD1, and RIPK2 with Western-blot and qPCR. BBR increased the expression of ATG12 and ATG16L1 and reduced the expression of NOD1 and RIPK2 in both RNA and protein levels (Figure 6B). RIPK2 had been reported to be able to regulate the production of lysozyme, and, after analyzing the results from transcriptomics and KEGG database, we speculated that BBR regulated the expression of lysozyme via the ATG16L1/NOD1/RIPK2 pathway as well (Figure 6A).

## 4. Discussion

It is widely believed that the gut is exposed to trillions of harmful antigens. Therefore, the intestinal defense system plays an important role in maintaining intestinal homeostasis. UC is a chronic recurrent intestinal inflammation with typical clinical features that include abdominal pain, diarrhea, and blood in the stool. UC cannot be cured but can only be ameliorated, and the disease process is often accompanied by onerous complications. Long-term colon inflammation increases the risk of colon cancer [31,32]. Although there are many treatments for UC, such as 5-aminosalicylic acid preparations, corticosteroids, thiopurines, biological agents, and surgery, serious side effects and inadequate treatments remain to be addressed [33,34]. Therefore, it is necessary to look for drugs with higher efficacy and fewer side effects. Our results indicated that BBR can alleviate the damage caused by DSS, and we also found that autophagy plays an important role in this process, because autophagy promoter (RAPA) can also ameliorate the situation of colitis, but autophagy inhibitor (3-MA) will attenuate the effect of BBR.

Paneth cells are important cells in the small intestine that provide the host with a defense reaction against microbial invasion. When bacteria or bacterial antigens invade the body, Paneth cells secrete antibacterial molecules such as defensins and lysozyme into the villi of the lumen of the small intestine to help maintain the gastrointestinal barrier [35]. Several IBD-associated genes can influence the function of Paneth cells, which indicates that Paneth cells are strongly associated with the pathogenesis of IBD [4]. At present, many important cellular activities in Paneth cells, such as autophagy and regulation of lysozyme, have not been fully understood yet [24,36]. 

Previous research has reported that in mice, intestinal bacteria induce the selective cargo classification of Paneth cells by interacting with NOD2 and LRRK2 proteins to promote symbiosis, and that NOD2 and LRRK2 were both encoded by genes associated with IBD [4]. The symbionts recruit NOD2 to gather in a dense-core vesicle (DCVs) containing lysozyme, which is necessary for DCV localization of LRRK2 and Rab2a [37]. NOD2, LRRK2, or Rab2a deficiency will lead to lysozyme degradation by lysosome [38]. However, whether there are other factors involved in the transportation of lysozyme and secretion, such as autophagy, is not known. We also set RAPA and BBR + 3-MA groups to verify if autophagy can influence the secretion of lysozyme in our DSS-induced mice model. Our results indicated that autophagy is associated with the expression and secretion of lysozyme. Autophagy can promote the maturation and production of lysosome in cells, and lysosome can degrade lysozyme. Therefore, we deduce that promoting autophagy may be the reason why BBR can decrease the secretion of lysozyme in Paneth cells. 

More and more studies have shown that autophagy plays an important role in the pathogenesis and progression of IBD. It has been reported that autophagy is closely related to a variety of gene mutations. Interestingly, autophagy-related 16-like 1 (Atg16L1) and NOD2, as the most representative IBD-related gene mutations, converge to participate in Paneth cell autophagy [39,40]. Because autophagy regulates the production and quality of lysosome in Paneth cells, impaired autophagy may cause the level of AMPs in the intestinal track to decrease, thus leading to the onset of IBD [36,41]. 

Excessive expression of lysozyme in the intestinal tract leads to a strong NOD signaling pathway, which in turn leads to excessive expression of proinflammatory factors, resulting in inflammatory damage [42]. NOD2 is a hot research area in recent studies, but we also examined NOD1, another important member of NLRs. NOD1 is widely expressed in IECs and laminar immune cells, and its expression in macrophages and IECs is important for inducing the immune response to bacteria and the production of antimicrobial peptides [43]. NOD1 can also activate the NF-κB and MAPK pathways to regulate inflammation reaction [44]. We examined the pathway of ATG16L1/NOD1/RIPK2 in IEC-18 and found that BBR can increase the expression of ATG16L1 and reduce the expression of NOD1 and RIPK2 in both RNA and protein levels. KEGG pathway analysis led us to the conclusion that RIPK2 can regulate lysozyme as well.

In conclusion, inhibiting the expression and secretion of lysozyme may be a new anti-inflammatory mechanism of BBR. The association between lysozyme and IBD should be further investigated.

## Data Availability

The data presented in this study are available on request from the corresponding author.

## Supplementary Materials

The following supporting information can be downloaded at: https://www.mdpi.com/article/10.3390/metabo12080676/s1. Table S1: Animal disease activity index (DAI) score sheet. Table S2: Mouse colon tissue injury score sheet. Table S3: Primer sequences used for the real-time PCR analysis.
