# Supplementary material for: Berberine Ameliorates Dextran Sulfate Sodium-Induced Ulcerative Colitis and Inhibits the Secretion of Gut Lysozyme via Promoting Autophagy
Source: Metabolites. 2022 Jul 23;12(8):676. doi: 10.3390/metabo12080676 (PMC9394306; doi:10.3390/metabo12080676)
Supplement: Supplementary file 1 [file metabolites-12-00676-s001.zip › metabolites-1825178-supplementary.pdf]

Table S1: Animal disease activity index (DAI) score sheet.

Percentage of weight loss		Stool shape		Stool bleeding	
Unchanged weight	0	Normal	0	Normal	0
1-5%	1	Loose stool	2	Occult blood	2
5-10%	2	Watery stool	4	Overt bleeding	4
10-15%	3				
>15%	4				

Table S2: Mouse colon tissue injury score sheet.

Histological changes in colon	Score
Normal histology	0
Histological damage is limited to endothelial cells, mild inflammatory cell infiltration	1
Focal ulcer changes, tissue structure destruction is limited to the mucosal layer, abnormal intestinal wall glandular structure, mild inflammatory cell infiltration	2
Focal, transmural ulcers and inflammation, mild to moderate inflammatory cell infiltration	3
Large transmural ulcers and inflammation, moderate inflammatory cell infiltration	4
Large ulcers and inflammation, lesions infiltrate from mucosa to serous membrane, severe inflammatory cell infiltration	5

Table S3: Primer sequences used for the real-time PCR analysis.

Gene name	Primer（5’-3’）	Temperature（℃）
AMPK	F: TCTGAGGGGCACCAAGAAAC<br>R: GTGGGTGTTGACGGAGAAGAG	60
ULK1	F: AAGTTCGAGTTCTCTCGCAAG<br>R: CGATGTTTTCGTGCTTTAGTTCC	60
LYZ1	F: GAGACCGAAGCACCGACTATG<br>R: CGGTTTTGACATTGTGTTCGC	60
DEFA1	F: TGTAGAAAAGGAGGCTGCAATAG<br>R: AGAACAAAAGTCGTCCTGAGC	60
CTSB	F: TCCTTGATCCTTCTTTCTTGCC<br>R: ACAGTGCCACACAGCTTCTTC	60
CTSD	F: GCTTCCGGTCTTTGACAACCT<br>R: CACCAAGCATTAGTTCTCCTCC	60
CTSL	F: GGGCTTTGTGAACGTCCAATC<br>R: CTGGAACGACTTGTGTTTAGTGT	60
DEFA5	F: CAGAGCAGCTACTAAGCGACT<br>R: AAAAGGGGAGATTCGGACAGA	60
CAMP	F: ATCCCGTACCACAAGCTCG<br>R: GGATGTGTAGGTGCTTCACTG	60
MTOR	F: TCCCCGGAACGAGGAACTC<br>R: TTCGCTCCACAGCCCATTTC	60
LC3B	F: GAAGGCACCCCATTGGGTT<br>R: AATCTCTGCATCTTCGGCTGA	60
GAPDH	F: TGGATTTGGACGCATTGGTC<br>R: TTTGCACTGGTACGTGTTGAT	60
